# Dissociating external power from intramuscular exercise intensity during intermittent bilateral knee‐extension in humans

**DOI:** 10.1113/JP274589

**Published:** 2017-09-02

**Authors:** Matthew J. Davies, Alan P. Benson, Daniel T. Cannon, Simon Marwood, Graham J. Kemp, Harry B. Rossiter, Carrie Ferguson

**Affiliations:** ^1^ School of Biomedical Sciences, Faculty of Biological Sciences & Multidisciplinary Cardiovascular Research Centre University of Leeds Leeds UK; ^2^ School of Exercise & Nutritional Sciences San Diego State University San Diego CA USA; ^3^ School of Health Sciences Liverpool Hope University Liverpool UK; ^4^ Magnetic Resonance & Image Analysis Research Centre University of Liverpool Liverpool UK; ^5^ Department of Musculoskeletal Biology University of Liverpool Liverpool UK; ^6^ Rehabilitation Clinical Trials Center, Division of Respiratory & Critical Care Physiology & Medicine Los Angeles Biomedical Research Institute at Harbor‐UCLA Medical Center Torrance CA USA

**Keywords:** bioenergetics, exercise intensity, exercise tolerance, interval exercise

## Abstract

**Key points:**

- Continuous high‐intensity constant‐power exercise is unsustainable, with maximal oxygen uptake (V˙O2 max ) and the limit of tolerance attained after only a few minutes.
- Performing the same power intermittently reduces the O_2_ cost of exercise and increases tolerance. The extent to which this dissociation is reflected in the intramuscular bioenergetics is unknown.
- We used pulmonary gas exchange and ^31^P magnetic resonance spectroscopy to measure whole‐body ${\dot{V}}_{O_{2}}$, quadriceps phosphate metabolism and pH during continuous and intermittent exercise of different work:recovery durations.
- Shortening the work:recovery durations (16:32 s *vs*. 32:64 s *vs*. 64:128 s *vs*. continuous) at a work rate estimated to require 110% peak aerobic power reduced ${\dot{V}}_{O_{2}}$, muscle phosphocreatine breakdown and muscle acidification, eliminated the glycolytic‐associated contribution to ATP synthesis, and increased exercise tolerance.
- Exercise intensity (i.e. magnitude of intramuscular metabolic perturbations) can be dissociated from the external power using intermittent exercise with short work:recovery durations.

**Abstract:**

Compared with work‐matched high‐intensity continuous exercise, intermittent exercise dissociates pulmonary oxygen uptake (${\dot{V}}_{O_{2}}$) from the accumulated work. The extent to which this reflects differences in O_2_ storage fluctuations and/or contributions from oxidative and substrate‐level bioenergetics is unknown. Using pulmonary gas‐exchange and intramuscular ^31^P magnetic resonance spectroscopy, we tested the hypotheses that, at the same power: ATP synthesis rates are similar, whereas peak ${\dot{V}}_{O_{2}}$ amplitude is lower in intermittent *vs*. continuous exercise. Thus, we expected that: intermittent exercise relies less upon anaerobic glycolysis for ATP provision than continuous exercise; shorter intervals would require relatively greater fluctuations in intramuscular bioenergetics than in ${\dot{V}}_{O_{2}}$ compared to longer intervals. Six men performed bilateral knee‐extensor exercise (estimated to require 110% peak aerobic power) continuously and with three different intermittent work:recovery durations (16:32, 32:64 and 64:128 s). Target work duration (576 s) was achieved in all intermittent protocols; greater than continuous (252 ± 174 s; *P *< 0.05). Mean ATP turnover rate was not different between protocols (∼43 mm min^−1^ on average). However, the intramuscular phosphocreatine (PCr) component of ATP generation was greatest (∼30 mm min^−1^), and oxidative (∼10 mm min^−1^) and anaerobic glycolytic (∼1 mm min^−1^) components were lowest for 16:32 and 32:64 s intermittent protocols, compared to 64:128 s (18 ± 6, 21 ± 10 and 10 ± 4 mm min^−1^, respectively) and continuous protocols (8 ± 6, 20 ± 9 and 16 ± 14 mm min^−1^, respectively). As intermittent work duration increased towards continuous exercise, ATP production relied proportionally more upon anaerobic glycolysis and oxidative phosphorylation, and less upon PCr breakdown. However, performing the same high‐intensity power intermittently *vs*. continuously reduced the amplitude of fluctuations in ${\dot{V}}_{O_{2}}$ and intramuscular metabolism, dissociating exercise intensity from the power output and work done.

## Key points

- Continuous high‐intensity constant‐power exercise is unsustainable, with maximal oxygen uptake (V˙O2 max ) and the limit of tolerance attained after only a few minutes.
- Performing the same power intermittently reduces the O_2_ cost of exercise and increases tolerance. The extent to which this dissociation is reflected in the intramuscular bioenergetics is unknown.
- We used pulmonary gas exchange and ^31^P magnetic resonance spectroscopy to measure whole‐body ${\dot{V}}_{O_{2}}$, quadriceps phosphate metabolism and pH during continuous and intermittent exercise of different work:recovery durations.
- Shortening the work:recovery durations (16:32 s *vs*. 32:64 s *vs*. 64:128 s *vs*. continuous) at a work rate estimated to require 110% peak aerobic power reduced ${\dot{V}}_{O_{2}}$, muscle phosphocreatine breakdown and muscle acidification, eliminated the glycolytic‐associated contribution to ATP synthesis, and increased exercise tolerance.
- Exercise intensity (i.e. magnitude of intramuscular metabolic perturbations) can be dissociated from the external power using intermittent exercise with short work:recovery durations.

**Abbreviations**
FID: free induction decay
L^−^: blood lactate
LT: lactate threshold
MR: magnetic resonance
MRS: magnetic resonance spectroscopy
P:O: oxygen cost of ATP resynthesis
P:W: ATP cost of force production
PCr: phosphocreatine
Pi: inorganic phosphate
pH_i_: intramuscular pH
RIT: ramp‐incremental test
${\dot{V}}_{O_{2}}$: oxygen uptake
V˙O2 max : maximal oxygen uptake
V˙O2 peak : peak oxygen uptake
V˙O2 SC : slow component of oxygen uptake

## Introduction

The coupling of internal (capillary‐to‐myocyte) to external (capillary‐to‐alveolus) O_2_ exchange during dynamic exercise is dependent on muscular oxidative ATP synthesis, the dynamics of the circulation, and volume of the intervening O_2_ stores, predominantly in the form of oxyhaemoglobin in the venous blood. At the onset of continuous constant‐power exercise, the kinetics of pulmonary oxygen uptake (${\dot{V}}_{O_{2}}$) are supplemented by contributions to energy transfer from utilization of O_2_ stores and, proportionally more significant, from substrate‐level phosphorylation [phosphocreatine (PCr) breakdown, glycolysis/glycogenolysis accumulating lactate]; termed the O_2_ deficit. The O_2_ deficit is associated with accumulation of products linked to muscle fatigue, such as intramuscular inorganic phosphate (Pi) and H^+^ (Allen *et al*. 2008), and hence ${\dot{V}}_{O_{2}}$ kinetics are strongly associated with exercise tolerance (Whipp & Ward 1992; Burnley & Jones, 2007; Sperandio *et al*. 2009; Murgatroyd *et al*. 2011): a fast response proffering greater exercise tolerance (Murgatroyd & Wylde, 2011; Rossiter, 2011).

${\dot{V}}_{O_{2}}$ kinetics are intensity‐dependent (Özyener *et al*. 2011). Critical power (the asymptote of the relationship between power and tolerable duration, which occurs between ∼60% and 80% V˙O2 max ; Poole *et al*. 1988; van der Vaart *et al*. 2014) marks the individual threshold in the rate of metabolic power production below which the bodily demands for ATP resynthesis are met by wholly‐aerobic energy transfer (Poole *et al*. 1988; Jones *et al*. 2008). During continuous exercise exceeding critical power, ${\dot{V}}_{O_{2}}$ continues to rise (through the action of the slow component; V˙O2 SC ), and intramuscular PCr breakdown and Pi and H^+^ accumulation are progressive (Poole *et al*. 1988; Jones *et al*. 2008; Vanhatalo *et al*. 2010). During constant power exercise above critical power, where duration exceeds ∼ 2 min (Hill *et al*. 2002), the limit of tolerance is commonly associated with the attainment of V˙O2 max , a minimum intramuscular [PCr] and intramuscular pH (pH_i_) and maximum [Pi] (Jones *et al*. 2008; Vanhatalo *et al*. 2010). This limits the volume of work that can be accumulated during constant power exercise above critical power (Monod & Scherrer, 1965; Moritani *et al*. 1981), where exercise can only be continued once a reduction in power to a value equal or below critical power is made (Gaesser & Poole, 1996; Coats *et al*. 2003; Ferguson *et al*. 2010).

Intermittent exercise, in which periods of supracritical power work are interspersed with periods of recovery, dissociates the work done from systemic (${\dot{V}}_{O_{2}}$ and blood lactate, [L^−^]) responses. Thus, the volume of work tolerated is increased, and the associated metabolic strain is reduced, using intermittent compared to continuous exercise (Astrand *et al*. 1960; Margaria *et al*. 1969; Turner *et al*. 2006; Combes *et al*. 2017). The magnitude of this mechanical‐to‐metabolic dissociation is dependent on the work:recovery duration, and is greatest when the work periods are short (e.g. 10–30 s) (Turner *et al*. 2006; Combes *et al*. 2017). The effect is that homeostasis of ${\dot{V}}_{O_{2}}$ and blood [L^−^] is less disturbed during intermittent compared to continuous exercise performed at the same power and accumulating the same volume of work. By the same notion, intermittent exercise can be used to provide a greater volume of supracritical power work in a given duration (Chidnok *et al*. 2013). This approach has been used in an attempt to enhance the stimulus for physiological adaptations by exercise training (Kemi *et al*. 2005; Helgerud *et al*. 2007; Wisløff *et al*. 2007; MacInnis *et al*. 2017).

It remains unclear the extent to which the systemic mechanical‐to‐metabolic dissociation by intermittent exercise (e.g. as frequently observed in pulmonary ${\dot{V}}_{O_{2}}$) (Turner *et al*. 2006; Guiraud *et al*. 2010; Chidnok *et al*. 2012; Combes *et al*. 2017) is matched by a similarly attenuated response of intramuscular phosphate metabolism. For example, intermittent exercise with short work bouts (10–30 s) and a low work:recovery ratio is hypothesized to have a relatively greater reliance on depletion of oxymyoglobin and venous oxyhaemoglobin O_2_ stores compared to exercise with longer work bouts (Astrand *et al*. 1960). This, coupled with a limb‐lung vascular transient delay, temporally dissociates cardiac output and O_2_ extraction responses at the lung, damping the response amplitude compared to the active muscles (Barstow & Mole, 1987; Barstow *et al*. 1990; Rossiter, 2011; Benson *et al*. 2013). This infers that intramuscular oxidative phosphorylation and PCr breakdown, and thus the intramuscular bioenergetic strain, would be increased (compared to the pulmonary ${\dot{V}}_{O_{2}}$ response) in short *vs*. longer intermittent work bouts or continuous exercise at the same power. However, this contradicts our knowledge of the progressive decrease in work efficiency during long intermittent or continuous exercise during which a V˙O2 SC  is observed, consequent to greater PCr breakdown resulting from increases in both intramuscular ATP cost of force production (P:W) and O_2_ cost of ATP production (P:O) (Rossiter *et al*. 2002; Krustrup *et al*. 2003; Turner *et al*. 2006; Bailey *et al*. 2010; Cannon *et al*. 2014).

We aimed to investigate the coupling dynamics of intramuscular bioenergetics to pulmonary gas exchange during continuous and intermittent exercise at the same power. We used ^31^P magnetic resonance (MR) spectroscopy (MRS) to measure intramuscular phosphate responses during bilateral knee‐extensor exercise in continuous and intermittent exercise of different work:recovery durations in comparison to pulmonary ${\dot{V}}_{O_{2}}$; each performed at the same power. We hypothesized that: (1) ATP synthesis rates are similar in intermittent and continuous exercise at the same power but (2) the peak pulmonary ${\dot{V}}_{O_{2}}$ amplitude will be lower in work‐matched intermittent *vs*. continuous exercise. Thus, we expect that: (3) intermittent exercise relies less upon anaerobic glycolysis for ATP provision than continuous exercise, and is associated with greater exercise tolerance; despite (4) short intervals requiring relatively greater fluctuations in intramuscular bioenergetics than in systemic pulmonary gas exchange compared to longer intervals.

## Methods

### Ethical approval

Liverpool Hope Faculty of Sciences and Social Sciences Research Ethics committee and the University of Liverpool Committee on Research Ethics approved the study, and all procedures complied with the latest version of the *Declaration of Helsinki*. Prior to participating all volunteers provided their written informed consent.

### Participants

Six healthy men (age: 24 ± 5 years; height: 176 ± 7 cm; weight: 80 ± 12 kg) (mean ± SD) volunteered to participate. All participants regularly undertook exercise, and any contraindications that would have precluded involvement in the study, including contraindications to MRS, were identified using a pre‐exercise assessment questionnaire.

### Exercise protocols

#### Ergometry

All exercise tests were performed on a computer‐controlled electromagnetically braked MR compatible bilateral knee‐extension ergometer (MRI Ergometer Up/Down; Lode BV, Groningen, The Netherlands). As described previously, this ergometer was customized for use in a 3T MR scanner (Siemens AG, Munich, Germany) using extended carbon‐fibre lever arms (Cannon *et al*. 2014). Participants lay prone with their feet secured into plastic stirrups using velcro straps. The stirrups were connected to the extended ergometer lever arms and attached to a drive crank for the electromagnetically braked flywheel. To isolate the work to the quadriceps, velcro strapping was also used to secure participants’ hips to the patient bed, minimizing contributions from the hip flexors and extensors. Using this ergometer, the external resistance is only applied during knee‐extension. The only work during knee‐flexion is that required to lift the mass of the lower leg. The range of motion is limited by the scanner dimensions to between ∼30° flexion and full extension. Participants were familiarized with performing a constant knee‐extension frequency of 90 kicks min^−1^ set using a metronome. This kick frequency also allowed the flywheel speed to be maintained above the minimum operating speed and aligned MR scanner acquisitions with muscle contractions.

#### Familiarization

The exercise protocols were completed in two phases: familiarization and testing. The familiarization phase took place in a temperature‐controlled human physiology laboratory. All exercise protocols began with a period of rest (∼1–3 min) and then knee‐extension exercise at 5 W (∼2–4 min), with each of these phases continued until a steady‐state was attained.

Participants first completed a ramp‐incremental exercise test (RIT) (3 W min^−1^) to the limit of tolerance, which was defined as the point at which the participant was unable to maintain the full range of motion at the target kicking frequency (90 kicks min^−1^) or when the flywheel speed decreased below the minimum operating speed, despite strong verbal encouragement. Participants were familiarized with the protocol by repeating it until the performance (power and duration) and physiological responses (${\dot{V}}_{O_{2}}$, etc.) were reproducible between visits (minimum of three repeats performed). Once familiarized, the power corresponding to 110% of RIT peak power was calculated and used in all subsequent exercise protocols. Comparison of V˙O2 peak  at the limit of RIT and continuous exercise was used to confirm V˙O2 max  (Poole & Jones, 2017). Continuous and intermittent protocols were also repeated until reproducible physiological responses were obtained (typically requiring two repeats).

#### Testing

The collection of pulmonary gas exchange data for matching to MRS data was performed in the same temperature‐controlled human physiology laboratory as the familiarization phase. Following a period of rest and warm‐up at 5 W, for the continuous exercise protocol, power was instantaneously applied at the power equivalent to 110% of RIT peak, and the participants were required to continue the exercise to the limit of tolerance. Intermittent protocols comprised periods of work at a power equivalent to 110% of RIT peak, and periods of recovery at 5 W. The three intermittent protocols performed by all participants had work:recovery durations of 16:32, 32:64 and 64:128 s. These durations were chosen to align with MRS data acquisition, with there being one ^31^P spectrum acquired every 8 s. Each intermittent protocol was continued until a total of 576 s of work was accumulated (at a 1:2 work:recovery duty cycle, this corresponded to a total duration of 28 min 48 s, allowing 216 complete ^31^P spectra to be collected), or to the limit of tolerance, whichever was the shorter. Only one exercise protocol was performed on a given day, with at least 24 h between visits, and protocols were performed in a random order.

Subsequently, continuous and intermittent exercise protocols were repeated inside the bore of a 3T superconducting magnet for measurement of intramuscular phosphate responses by ^31^P MRS using the same ergometer and the same exercise protocol as used for pulmonary gas exchange data collection.

### Pulmonary gas exchange

Participants breathed through a facemask for measurement of respired gases (Zan 600; Geratherm, Geschwenda, Germany). Volume and flow rates were sampled at 125 Hz and measured using a pneumotach, with O_2_ and CO_2_ gas concentrations being measured using electrochemical cell and infrared gas analysers, respectively. Using BlueCherry software (Geratherm), gas concentration and volume signals were time‐aligned for online calculation of breath‐by‐breath pulmonary gas exchange and ventilatory variables.

Prior to each test, the flow sensor and gas analysers were calibrated in accordance with the manufacturers’ guidelines. The pneumotach was calibrated using a 3 litre syringe across a range of flow rates, with the gas analysers calibrated using certified gas mixtures that spanned the expected inspired and expired ranges of both O_2_ and CO_2_.

### ^31^P magnetic resonance spectroscopy

Relative concentrations of intramuscular phosphates (ATP, PCr, Pi) were measured using a 3T superconducting magnet (Magnetom Trio; Siemens AG) and pH_i_ was calculated from the chemical shift of Pi to PCr (Moon & Richards, 1973). A one‐pulse ^31^P MRS acquisition was employed using a dual‐tuned (^1^H, 15 cm diameter; ^31^P 18 cm diameter) surface radiofrequency coil (RAPID Biomedical GmbH, Rimpar, Germany) placed under the knee‐extensors of the right leg and positioned halfway between the hip and knee. This provided a metabolic signal from a mid‐thigh slice of the rectus femoris, vastus medialis, vastus intermedialis and vastus lateralis (Cannon *et al*. 2014). Once in the correct position, the hips of participants were secured to the scanner bed using non‐distensible velcro straps. Participants were then moved inside the bore of the magnet and the scanning procedure commenced.

Sagittal and coronal gradient‐recalled echo images of the thigh were taken to confirm placement of the radiofrequency coil in relation to the knee‐extensors. ^1^H shimming was performed to optimize magnetic field homogeneity. Subsequently, a fully relaxed high‐resolution unsaturated spectrum and 32‐scan spectrum (repetition time of 10 s) were obtained, with this being used as the reference baseline spectra. Throughout the protocol, ^31^P free induction decays (FIDs) were collected every 2 s, with four FIDs used to provide a spectrum every 8 s. The continuous and intermittent exercise protocols were aligned to ensure that each spectrum did not straddle work‐recovery transitions.

### Data analysis

All breath‐by‐breath ${\dot{V}}_{O_{2}}$ responses were filtered to remove any erroneous breaths (defined as those occurring outside the 99% prediction limits of the local mean) resulting from sighs, coughs or swallowing, etc. (Lamarra *et al*. 1987). For the RIT, lactate threshold (LT) was estimated non‐invasively using standard ventilatory and pulmonary gas exchange criteria (Whipp *et al*. 1986). In both RIT and continuous constant‐power exercise, V˙O2 peak  was identified as the greatest 12‐breath (∼20 s) moving average prior to the limit of tolerance.

**Figure 1:**
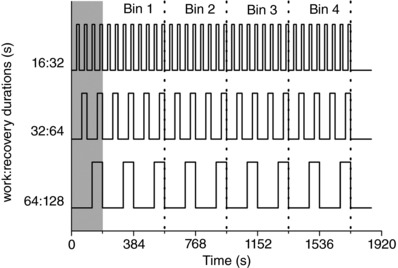
Schematic of the intermittent exercise protocols and time‐bins used for ${\dot{V}}_{O_{2}}$ and ^31^P MRS measures Following a warm‐up at 5 W, intermittent exercise with work phases performed at 110% of ramp‐incremental peak power was initiated with work:recovery durations of either 16:32 s (top), 32:64 s (middle) or 64:128 s (bottom). The first 192 s of each test was eliminated (grey box) to exclude a kinetic transient phase that preceded the stabilization of ${\dot{V}}_{O_{2}}$ and ^31^P MRS fluctuations, with like transitions in each time‐bin time‐aligned to exercise onset and data averaged to improve signal:noise.

For the intermittent responses, breath‐by‐breath data were linearly interpolated to provide a value every second. ${\dot{V}}_{O_{2}}$ data were then phase‐aligned to PCr to account for the limb‐lung vascular transit delay (Rossiter *et al*. 1999) and then averaged to provide a datum every 8 s (i.e. to match the intervals of ^31^P data collection). Intermittent exercise was characterized by an expected transient phase where the amplitude of the work‐recovery fluctuations in ${\dot{V}}_{O_{2}}$ were climbing (the first 192 s in the present study) and a subsequent periodic steady‐state phase where the amplitude of ${\dot{V}}_{O_{2}}$ fluctuations stabilized between exercise and recovery phases. For this reason, to analyse the time course of the intramuscular and pulmonary responses to intermittent exercise, the first 192 s were eliminated and the subsequent ${\dot{V}}_{O_{2}}$ and phosphate data sorted into time‐bins of 384 s each, resulting in a total of four repeats (or bins) of intermittent work‐recovery phases (Fig. 1). Within each time‐bin, like transitions were aligned to the onset of work at 110% of RIT peak power and averaged to increase the signal:noise (Lamarra *et al*. 1987; Rossiter *et al*. 2000). The peak, nadir and peak‐to‐nadir amplitude of fluctuations in each variable were identified within each bin. All data were then normalized to the amplitudes measured during continuous exercise between 5 W (0%) and peak (100%).

### Kinetic analysis of ^31^P MRS data

This procedure has been described previously (Cannon *et al*. 2014). Briefly, PCr kinetics were modelled using non‐linear least‐squares regression (implemented in Excel; Microsoft Corp., Redmond, WA, USA). The rate of ATP turnover was estimated from the contributions of PCr breakdown (*D*), oxidative phosphorylation (*Q*) and glycogenolysis (*L*), which were determined from the PCr, Pi and pH_i_ data acquired during exercise and recovery, using methods described in detail elsewhere (Kemp, 2015, 2016). To improve the signal:noise, ATP turnover was calculated as a mean rate throughout the work phases of the protocols (i.e. mean of the four bins).

Estimating ATP turnover using ^31^P MRS *in vivo* relies on some assumptions, particularly in relation to the estimated contribution of oxidative phosphorylation (*Q*) (Kemp, 2015, 2016). However, sensitivity analysis suggests that none of the calculations used depended substantially on any particular assumption. Using the initial PCr breakdown rate (*D*) as a measure of initial ATP turnover, and initial recovery PCr resynthesis as a measure of end‐exercise supra‐basal oxidative ATP synthesis rate (*Q*) depends on only the most general of assumptions about closed‐loop feedback control of oxidative ATP synthesis; the use of the relationship between *Q* and [ADP] established by analysis of recovery kinetics to ‘predict’ *Q* during exercise assumes only one of several possible modes of mitochondrial feedback control (Kemp, 2015), which each provide very similar results during exercise of this type. Finally, the calculated contribution of glycolytic ATP production is small in the present study and depends on uncontroversial models of cellular pH buffering, as well as assumptions of approximately linear pH dependence of acid efflux to which the detailed results are rather insensitive (Kemp, 2015, 2016).

### Statistical analysis

Metabolic perturbations (peak, nadir and peak‐to‐nadir amplitude) were initially compared among the four time‐bins using a one‐way repeated measures ANOVA, to investigate the effect of time on metabolic disturbances. Subsequently, peak continuous exercise values and final time‐bin values for all intermittent protocols were compared using a one‐way repeated measures ANOVA to investigate the effect of exercise protocol (continuous and three intermittent protocols) on metabolic disturbances. Finally a two‐way repeated measures ANOVA was used to compare the relative amplitude of change (${\dot{V}}_{O_{2}}$ *vs*. PCr), and to investigate how this changed between intermittent protocols (16:32 *vs*. 32:64 *vs*. 64:128 s). *Post hoc* Tukey‐corrected pairwise comparisons were performed where appropriate. *P* < 0.05 was considered statistically significant. All values are reported as the mean ± SD.

## Results

### Ramp incremental responses

The estimated LT was 1.46 ± 0.26 L min^−1^ (72 ± 2% V˙O2 peak ), with the tolerable limit attained at a V˙O2 peak  of 2.04 ± 0.36 L min^−1^ and peak power of 34 ± 7 W.

### ATP turnover and exercise tolerance during continuous and intermittent exercise

**Figure 2:**
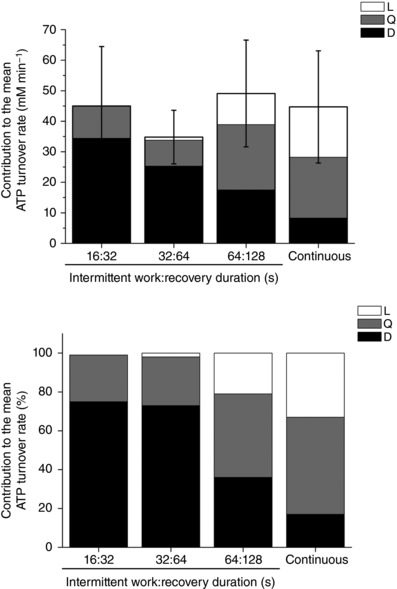
Contributions from phosphocreatine breakdown (*D*), oxidative phosphorylation (*Q*) and anaerobic glycolysis (*L*) to the mean ATP turnover rate at 110% of ramp‐incremental peak power during continuous and intermittent exercise comprising work:recovery durations of 16:32, 32:64 and 64:128 s Upper: absolute energetic system contributions to mean ATP turnover. Lower: relative energetic system contributions to mean ATP turnover.

Continuous constant‐power exercise at 110% RIT peak power (38 ± 7 W) was sustained for 252 ± 174 s and V˙O2 peak  at the limit of tolerance (2.03 ± 0.26 L min^−1^) was not different from RIT V˙O2 peak , confirming V˙O2 max  (*P* = 0.891). The mean rate of ATP turnover during continuous exercise performed to the limit of tolerance was 44.7 ± 18.4 mm min^−1^, with large contributions from anaerobic glycolysis (*L*; 33 ± 19%) and oxidative phosphorylation (*Q*; 50 ± 23%) compared to PCr breakdown (*D*; 17 ± 6%) (Fig. 2 and Table 1). At intolerance in continuous exercise, PCr declined to 38 ± 13% of baseline and pH_i_ reached a nadir of 6.67± 0.07 (*vs*. 7.07 ± 0.04 at rest).

**Table 1:** Mean ATP turnover, and contributions from phosphocreatine breakdown (*D*), oxidative phosphorylation (*Q*) and anaerobic glycolysis (*L*) during continuous and intermittent bilateral knee‐extension exercise

	ATP	*D*	*Q*	*L*
Protocol	mm min^−1^	mm min^−1^	mm min^−1^	mm min^−1^
Continuous	44.7 ± 18.4	8.3 ± 5.7†, ‡	19.9 ± 8.8†, ‡	16.4 ± 14.4†, ‡
16:32	45.0 ± 19.5	34.4 ± 15.7*, §	10.5 ± 4.8*, §	0.1 ± 0.0*
32:64	34.8 ± 8.8	25.3 ± 6.1*	8.5 ± 2.5*, §	1.0 ± 1.7*
64:128	49.1 ± 17.5	17.5 ± 6.1†	21.4 ± 9.8†, ‡	10.2 ± 4.3

Continuous exercise was performed to the limit of tolerance (252 ± 174 s). Intermittent exercise was performed with work:recovery durations of 16:32, 32:64 and 64:128 s, each for a total duration of 28 min 48 s.

Values are presented as the mean ± SD. ^*^ *P* < 0.05 *vs*. continuous; ^†^ *P* < 0.05 *vs*. 16:32 s intermittent exercise; ^‡^ *P* < 0.05 *vs*. 32:64 s intermittent exercise; ^§^ *P* < 0.05 *vs*. 64:128 s intermittent exercise.

**Figure 3:**
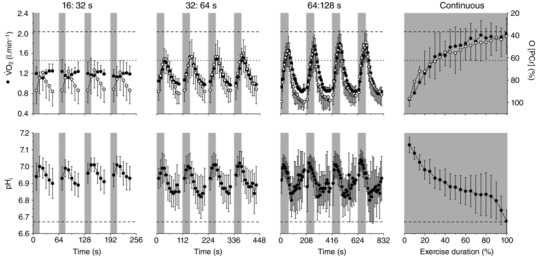
${\dot{V}}_{O_{2}}$, PCr (top row) and pH_i_ (bottom row) responses to work:recovery durations of 16:32 s (first column), 32:64 s (second column), 64:128 s (third column) or continuous exercise (forth column) Also shown is the lactate threshold (LT) from the ramp‐incremental exercise test (dotted line), as well as the V˙O2 max  (top row, dashed line) and pH_i_ (bottom row, dashed line) attained at the limit of tolerance of the continuous exercise protocol. Grey areas indicate the exercise period performed at 110% of ramp incremental peak power. Note, in the 16:32 s protocol, that ${\dot{V}}_{O_{2}}$ never exceeds the LT, and there are only minor changes in pH_i_, consistent with the 16:32 s intermittent protocol being moderate‐intensity. The peak ${\dot{V}}_{O_{2}}$ amplitude exceeds the LT in the 32:64 and 64:128 s intermittent protocols and during continuous exercise, with this accompanied by a metabolic acidosis (decline in pH_i_), consistent with a greater exercise metabolic strain in these protocols.

In all intermittent protocols, the 576 s target of work at 110% RIT peak power was accumulated. This equated to 327 ± 180% more work performed during intermittent exercise than with continuous exercise at the same power. Mean ATP turnover was not different among continuous and the work phases of intermittent exercise protocols (*P* > 0.05) (Table 1). Following removal of the initial kinetic phase (first 192 s), the four binned‐repeats of the work‐recovery phases of intermittent exercise did not differ (*P* > 0.05) within the 16:32 s or 32:64 s intermittent protocols. In other words, the ${\dot{V}}_{O_{2}}$, PCr and pH_i_ fluctuation peak, fluctuation nadir and fluctuation amplitude were constant following the removal of the initial 192 s kinetic phase (Fig. 3). However, for the 64:128 s intermittent protocol, peak metabolic disturbance (PCr; *P* < 0.05) and fluctuation amplitude (${\dot{V}}_{O_{2}}$; *P* < 0.05) increased between time‐bins 1 and 4. For these reasons, the ${\dot{V}}_{O_{2}}$, PCr and pH_i_ peak values used for all subsequent analyses were those from the final bin of intermittent exercise in all protocols (i.e. the values measured in the fourth time‐bin of Fig. 1).

### Absolute bioenergetic and pulmonary responses during continuous and intermittent exercise

When comparing within variables across the four different exercise protocols, the absolute ${\dot{V}}_{O_{2}}$ increase, PCr breakdown and pH_i_ fall were less during short work:recovery intermittent exercise *vs*. long work:recovery duration exercise (*P* < 0.05) (Fig. 3 and Table 2). The peak values of the disturbance in ${\dot{V}}_{O_{2}}$, PCr and pH_i_ during the 16:32 s intermittent protocol did not reach those seen during continuous exercise (*P* < 0.05). Similarly, the peak values of the disturbance in ${\dot{V}}_{O_{2}}$ and pH_i_ during the 32:64 s intermittent protocol were less than those during continuous (*P* < 0.05), although peak PCr was not different (*P* = 0.07). However, the absolute peak values of the disturbance of ${\dot{V}}_{O_{2}}$ (*P* = 0.06), PCr (*P* = 0.72) and pH_i_ (*P* = 0.08) during 64:128 s intermittent exercise were not different from those at the limit of tolerance in continuous exercise (Fig. 3 and Table 2).

**Table 2:** Absolute and relative [normalized between 5 W baseline (0%) and the limit of tolerance during continuous exercise (100%)] peak metabolic responses during continuous and intermittent exercise at 110% of ramp incremental peak power

		Continuous	Intermittent exercise		
		exercise	16:32	32:64	64:128
${\dot{V}}_{O_{2}}$	L min^−1^	2.03 ± 0.26	1.28 ± 0.24*, ‡, §	1.54 ± 0.36*, †, §	1.80 ± 0.31†, ‡
	% Continuous	100 ± 0	45.1 ± 7.0§	63.7 ± 14.8§	83.6 ± 13.1†, ‡
PCr	% Baseline	38.2 ± 13.0	73.1 ± 16.2*, §	55.8 ± 16.5	45.0 ± 15.8
	% Continuous	0 ± 0	54.4 ± 27.2‡, §	29.9 ± 21.7†	9.6 ± 25.3†
pH_i_		6.67 ± 0.07	6.92 ± 0.07*, §	6.84 ± 0.12*	6.77 ± 0.12†
	% Continuous	100 ± 0	38.4 ± 11.3§	60.0 ± 23.8	77.5 ± 31.2

Continuous exercise was performed to the limit of tolerance (252 ± 174 s). Intermittent exercise was performed with work:recovery durations of 16:32, 32:64 and 64:128 s, each for a total duration of 28 min 48 s.

Values are presented as the mean ± SD. ^*^ *P* < 0.05 *vs*. continuous; ^†^ *P* < 0.05 *vs*. 16:32 s intermittent exercise; ^‡^ *P* < 0.05 *vs*. 32:64 s intermittent exercise; ^§^ *P* < 0.05 *vs*. 64:128 s intermittent exercise.

### Relative fluctuations in intramuscular bioenergetics and pulmonary ${\dot{V}}_{O_{2}}$ during intermittent compared to continuous exercise

To compare the relative excursion between intramuscular and pulmonary variables, responses were normalized between 5 W baseline and peak values of continuous exercise. Comparing between ${\dot{V}}_{O_{2}}$ and PCr during intermittent exercise, the relative peak to nadir amplitude of ${\dot{V}}_{O_{2}}$ and PCr fluctuations increased with work bout duration (*P* < 0.05) (Table 3), with a strong inverse relationship between PCr breakdown and ${\dot{V}}_{O_{2}}$ (*r* ^2^ = 0.88; *P* < 0.05). However, the amplitude of the ${\dot{V}}_{O_{2}}$ fluctuation was less than that of PCr for 16:32 and 32:64 s protocols (*P* < 0.05) (Fig. 3 and Table 3). The relative contribution of PCr breakdown to intramuscular ATP production was greatest during the short intermittent cycles (16:32 and 32:64 s). At the longer cycles (64:128 s and continuous), the contributions from oxidative phosphorylation (*Q*) and anaerobic glycolysis (*L*) were at their greatest (*P* < 0.05) (Fig. 2).

**Table 3:** The relative amplitudes of ${\dot{V}}_{O_{2}}$, PCr and pH_i_ fluctuations during intermittent bilateral knee‐extension exercise compared with continuous exercise

work:recovery duration	${\dot{V}}_{O_{2}}$ (%)	PCr (%)	pH_i_ (%)
16:32	17.0 ± 6.9	32.1 ± 20.6*	21.3 ± 7.7
32:64	41.3 ± 15.0†	60.2 ± 12.5*, †	48.3 ± 23.9†
64:128	77.2 ± 18.1†, ‡	85.7 ± 21.3†, ‡	74.4 ± 30.3†, ‡

Values are normalized between 5 W baseline (0%) and the limit of tolerance during continuous exercise (100%). Power is 110% of ramp incremental peak power. Continuous exercise was performed to the limit of tolerance (252 ± 174 s). Intermittent exercise was performed with work:recovery durations of 16:32, 32:64 and 64:128 s, each for a total duration of 28 min 48 s.

Values are presented as the mean ± SD. ^*^ *P* < 0.05 between ${\dot{V}}_{O_{2}}$ and PCr in the same exercise protocol. Within variables (i.e. within ${\dot{V}}_{O_{2}}$, PCr or pH_i_): ^†^ *P* < 0.05 from the 16:32 s intermittent protocol; ^‡^ *P* < 0.05 *vs*. both 16:32 and 32:64 s intermittent protocols.

## Discussion

The major finding of the present study was that the metabolic strain of exercise (${\dot{V}}_{O_{2}}$, intramuscular PCr breakdown, pH_i_) is dissociated from the external power and cellular demand for ATP production by performing the exercise intermittently. Although continuous constant‐power exercise at 110% peak RIT power could only be sustained for ∼4 min, our findings are consistent with previous studies reporting that exercise tolerance was increased by at least 3‐fold, and a greater volume of work accumulated, when the same power is performed intermittently (Astrand *et al*. 1960; Margaria *et al*. 1969; Turner *et al*. 2006; Chidnok *et al*. 2013; Skiba *et al*. 2014). We found that mean ATP turnover during the work phases was not different for both continuous and intermittent exercise at the same external power (Table 1), such that alterations in work efficiency could not explain the differences in tolerance. Nevertheless, the magnitude of intramuscular metabolic fluctuations was attenuated during intermittent exercise. This dissociation was greatest when the work:recovery durations were shorter (Fig. 3), despite the work:recovery duty cycle (1:2) and power output remaining constant for all intermittent protocols.

These data support our hypotheses that ATP synthesis rates would be similar in intermittent and continuous exercise at the same external power (110% peak RIT power; hypothesis 1), despite pulmonary ${\dot{V}}_{O_{2}}$ fluctuations being lower in intermittent exercise (hypothesis 2). We also found, in contrast to some suggestions (Rossiter *et al*. 2002; Krustrup *et al*. 2003; Cannon *et al*. 2014), that the small fluctuations in pulmonary ${\dot{V}}_{O_{2}}$ during the shorter *vs*. longer work:recovery durations, were not mirrored in the intramuscular responses. As intermittent work interval duration increased towards matching the continuous protocol, the mean ATP production relied increasingly upon anaerobic glycolysis and oxidative phosphorylation and less upon PCr breakdown (hypothesis 3). On the other hand, during short work:recovery intermittent exercise, the relative amplitude of the ${\dot{V}}_{O_{2}}$ fluctuations were damped compared to those of intramuscular PCr (hypothesis 4): The ratio between relative amplitudes of ${\dot{V}}_{O_{2}}$ and PCr fluctuations were 53% during 16:32 s, 69% during 32:64 s, rising to 90% during 64:128 s (Fig. 3 and Table 3). This is consistent with proportionally greater contributions to the ATP turnover from PCr hydrolysis and suggests proportionally greater stored O_2_ usage during short work:recovery intermittent exercise than longer work:recovery intermittent exercise or continuous constant‐power exercise (Fig. 2) (Turner *et al*. 2006). It also suggests that the capacitance of the intervening energy and O_2_ stores has a significant impact in damping the external (pulmonary) respiratory responses to intermittent exercise relative to the internal (intramuscular) bioenergetics.

### Intermittent exercise tolerance

At the onset of continuous exercise, the ability of intramuscular oxidative phosphorylation to meet the cellular ATP requirement is dependent on its kinetics, with any shortfall compensated for by substrate‐level phosphorylation (O_2_ deficit). This non‐oxidative ATP supply is capacity‐limited and propagates a ‘fatigue cascade’ (Murgatroyd & Wylde, 2011). This cascade leads to the accumulation of fatigue‐related metabolites, exercise inefficiency (reflected in the V˙O2 SC ), intramuscular PCr depletion and, ultimately, exercise intolerance (Jones *et al*. 2008; Vanhatalo *et al*. 2010). Consequently, the rate at which intramuscular oxidative phosphorylation responds to alterations in ATP demand (${\dot{V}}_{O_{2}}$ kinetics) is a key determinant of high‐intensity exercise tolerance (Whipp & Ward, 1992; Jones & Burnley, 2009; Murgatroyd *et al*. 2011). Mean ATP turnover was not different between protocols (Table 1) and therefore the initial rate of ${\dot{V}}_{O_{2}}$ change was the same at the onset of both continuous and intermittent exercise regardless of work:recovery duration (DiMenna *et al*. 2010). Consequently, the amplitude of the intramuscular ${\dot{V}}_{O_{2}}$ fluctuation, as well as the requirement for substrate‐level phosphorylation, was determined by the intermittent work duration. Although shortening the intermittent duration resulted in a relatively greater proportional contribution by PCr breakdown to overall ATP synthesis, it also resulted in increased system stability and exercise tolerance. Accordingly, ${\dot{V}}_{O_{2}}$, PCr and pH_i_ fluctuations were small and there was no measurable cellular contribution to the exercise task from anaerobic glycolysis. Indeed, the ${\dot{V}}_{O_{2}}$ fluctuations during the shortest intermittent protocol remained below the estimated lactate threshold throughout. This cellular bioenergetics response is consistent with the observations that exercise was better sustained, and more work done, during intermittent compared to continuous exercise.

### Damping of pulmonary respiration by cellular bioenergetics

During short work:recovery intermittent exercise the peak fluctuation in ${\dot{V}}_{O_{2}}$ *vs*. PCr (17.0 ± 6.9 *vs*. 32.1 ± 20.6%) suggests that the relative intramuscular metabolic strain is greater than that extrapolated from the ${\dot{V}}_{O_{2}}$ measured at the mouth. The dissociation between muscle ${\dot{V}}_{O_{2}}$ (inferred from PCr) and pulmonary ${\dot{V}}_{O_{2}}$ (measured) during short work bouts is probably a result of rapid transients in intramuscular and venous O_2_ storage. The ∼10 s delay after the onset of high‐intensity exercise in the appearance of deoxygenated myoglobin (Richardson *et al*. 2015) suggests that venous haemoglobin deoxygenation (Turner *et al*. 2006) bears the brunt of this damping process (Astrand *et al*. 1960) and may result in a narrowing of the capillary‐to‐myocyte $P_{O_{2}}$ (partial pressure of oxygen) driving pressure. This finding is also consistent with slow activation of muscle oxidative phosphorylation at exercise onset (Korzeneiski & Rossiter, 2015). Given that the ${\dot{V}}_{O_{2}}$ in the present study was measured at the mouth without use of an algorithm to estimate alveolar gas exchange, there is also the potential for a contribution from changes in pulmonary O_2_ stores (Beaver *et al*. 1981; Aliverti *et al*. 2004; Wüst *et al*. 2008). Although the degree of this effect is unknown, any changes in end‐expiratory lung volume are anticipated to be small during this prone exercise task.

### Dissociating exercise intensity from power output

The phrases ‘exercise intensity’ and (relative) ‘power output’ are commonly used interchangeably. The finding that intensity and power output can be completely dissociated depending on the work:recovery duration highlights the importance of providing these two terms with distinct definitions. The dissociation here occurred to the degree that a severe intensity exercise bout (where ${\dot{V}}_{O_{2}}$ exceeded critical power) could be reduced to moderate intensity (where ${\dot{V}}_{O_{2}}$ remained below the lactate threshold) through shortening the duration of work intervals, despite the power output and total work done remaining constant. Thus, the term power output refers to a rate of energy transfer from the skeletal muscle to perform external work (mechanical power), whereas the intensity that a given power output engenders depends on the peak magnitude of the metabolic fluctuation(s) evoked during the task. By shortening the work:recovery durations, intensity (including the requirement for anaerobic glycolysis to contribute to the ATP turnover) is minimized and exercise better sustained.

In the present study, the fluctuation in the ${\dot{V}}_{O_{2}}$ response to intermittent exercise was considerably damped compared to intramuscular PCr. Nevertheless, in the short‐duration intermittent protocol (16:32 s), where the magnitude of this effect was greatest, there remained a large dissociation between the external power and the intramuscular metabolic strain. This was achieved by terminating the work bout before intramuscular PCr substantially decreased, and allowing PCr to increase during the intervening recovery interval. During the shortest work:recovery duration of intermittent exercise, we found that the peak and nadir of the ${\dot{V}}_{O_{2}}$ and PCr fluctuations remained below values associated with the lactate threshold and there were no net contributions from anaerobic glycolysis to meet the cellular demands for ATP turnover, despite power exceeding that achieved at V˙O2 max  in the RIT. This bioenergetics behaviour is consistent with responses observed during continuous exercise at far lower powers that are termed moderate intensity (Wasserman *et al*. 1967; Rossiter *et al*. 2002). The accumulation of lactate and the associated intramuscular acidosis occurs relatively slowly after exercise onset (e.g. glycolysis itself is not activated for ∼10–15 s after exercise onset) (Conley *et al*. 1998; Walsh *et al*. 2008). However, any delayed activation of glycolytic flux is unlikely to be a major contributor to the relative preservation of muscle pH_i_ and lack of muscle acidification in this protocol because the 16 s exercise bout was repeated many times over the ∼30 min protocol; which would certainly be sufficient to identify any activation of glycolytic flux. The strong probability is that any cytosolic redox challenge consequent to increased glycolytic flux was met either by intramitochondrial transport of accumulated pyruvate (effectively reversing any lactate formation during the work bout), or of NADH^+^, during the recovery phases of the intermittent bouts. Because sustained energy provision was not required, the very short work bouts and interspersed recovery intervals allowed aerobic energy provision to remain below the lactate threshold and the substrate‐level contributions to the exercise energetics in short intermittent work bouts appear to be essentially limited to PCr breakdown (Fig. 2).

We also observed (Fig. 3) that, during the work phases of intermittent exercise, pH_i_ increases when PCr is falling (as H^+^ is sequestered in the Lohmann reaction: ADP + PCr + H^+^ ←→ ATP + Cr). This means that, during short intermittent bouts, the lowest pH_i_ occurs during recovery where PCr is greatest and the muscle is alkalotic during the work phase when PCr is lowest. This is unlike during the longer duration intermittent bouts (64:128 s) or continuous exercise, where PCr and pH_i_ are both low during the muscular activity. Whether this alkalinizing effect during short intermittent exercise is protective of muscle fatigue is currently unclear, although clearly the lesser magnitude of PCr breakdown (and Pi accumulation) is associated with increased exercise tolerance and a prolongation of work capacity. Furthermore, the influence of this effect on the cellular transduction of training responses is currently unknown (see below).

Extending the work:recovery durations predictably increased the intramuscular metabolic strain. In the 32:64 s protocol, the peak ${\dot{V}}_{O_{2}}$ fluctuation (1.54 ± 0.36 L min^−1^) exceeded the estimated lactate threshold (1.46 ± 0.26 L min^−1^), which was associated with a cellular acidosis (pH_i_; 6.84 ± 0.12) and an increased contribution from anaerobic glycolysis to ATP turnover. These features are consistent with heavy‐intensity exercise (where metabolic power production is between the lactate threshold and critical power). The sustained decrease in pH_i_ in the 32:64 s protocol demonstrates that the O_2_ deficit accumulated during the work phase to the extent that anaerobic glycolysis became a necessary contributor to the energy transfer (Fig. 2). The magnitudes of the intramuscular energetic strain and acidosis are consistent with those in continuous exercise at a power just below critical power (estimated to be ∼60–80% peak aerobic power during cycle ergometry) (Wasserman *et al*. 1967; Rossiter *et al*. 2002; Jones *et al*. 2008). Again, the peak intramuscular acidosis occurred during recovery, rather than during the work phase of the intermittent exercise. Our data emphasize that it is not the mean metabolic response during intermittent exercise, but rather the peak of the metabolic perturbation that is probably important in determining the intramuscular metabolic strain: the mean ${\dot{V}}_{O_{2}}$ during the 32:64 s intermittent protocol was below the lactate threshold (1.18 ± 0.17 *vs*. 1.46 ± 0.26 L min^−1^), which reflects an average of the entire work:recovery cycle.

We would expect the sustained metabolic acidosis during the 32:64 s intermittent protocol to be associated with a slow component in both ${\dot{V}}_{O_{2}}$ and PCr. However, there was no progressive increase in ${\dot{V}}_{O_{2}}$ and decrease in PCr between time bins during either 16:32 s or 32:64 s protocols. This, together with no difference in mean ATP turnover rate among protocols, suggests that there was no change in either the efficiency of force production (P:W) or mitochondrial efficiency (P:O) during the acidifying heavy‐intensity intermittent protocol. This has implications for work efficiency and the mechanisms contributing to the V˙O2 SC . Work efficiency is typically assumed constant during the early transient (e.g. first 60 s) of either sub‐ or supra‐LT exercise. However, findings in stimulated dog muscle (Wust *et al*. 2011) and in some human studies (Bangsbo *et al*. 2001; Koppo *et al*. 2004) suggest that work efficiency may be initially high and rapidly decline over the first ∼15–30 s of contraction before rebounding and levelling out after ∼1–2 min. For exercise above LT, a second decline in work efficiency is observed after ∼2 min as the V˙O2 SC  develops. Our data that ATP turnover appeared greater at 16:32 s compared to 32:64 s (albeit non‐significant) may reflect some effect of rapid changes in work efficiency in the very early transient. Subsequently, for the longer intermittent and the continuous protocol, work inefficiencies associated with the V˙O2 SC  became increasingly evident. We speculate that, as the peak of the metabolic fluctuation in the 32:64 s protocol only exceeded the LT for a few seconds (∼8 s, on average) at the end of each work phase, the intervening recovery was sufficient to constrain any transient fatiguing processes that contribute to the V˙O2 SC . Without the accumulation of muscle fatigue, the drive for progressive work inefficiency in the form of a ${\dot{V}}_{O_{2}}$ or PCr slow component was absent (Cannon *et al*. 2011; Grassi *et al*. 2015; Keir *et al*. 2016). Although prolonging the work:recovery duration increased the magnitude of metabolic perturbations and exercise intensity above that seen during 16:32 s, there was still a clear dissociation between the external mechanical power and the exercise intensity (intramuscular metabolic strain).

During exercise with the longest work:recovery (64:128 s) protocol, there was an increase in the intramuscular strain (Figs 2 and 3). The peak intramuscular responses during the 64:128 s intermittent protocol were consistent with those during continuous exercise above critical power (Jones *et al*. 2008). A progressive reduction in work efficiency was present, with the ${\dot{V}}_{O_{2}}$ and PCr fluctuations in the final work phases (bin 4) (Figs 1 and 3) exceeding those of the first work phase (bin 1; *P* < 0.05). Despite this, we did not observe this effect in the ATP turnover rate during the 64:128 s intermittent protocol. This may be influenced by the necessity to calculate ATP turnover as the mean rate of the work phases to increase signal:noise, which also reduced the ability to detect an inefficiency by this method. The reduction in work efficiency (as reflected in the ${\dot{V}}_{O_{2}}$ and PCr responses) (Fig. 3) is probably consequent to an increase in the ATP requirement to maintain power production (Cannon *et al*. 2014). Although the mechanism(s) responsible for a progressive reduction in work efficiency during the V˙O2 SC  remain controversial, the prevailing suggestion during voluntary exercise is that progressive recruitment of motor units innervating low oxidative and/or type II muscle fibres may be responsible (Pringle *et al*. 2003; Krustrup *et al*. 2004). Although a reduction in the mitochondrial P:O has yet to be completely ruled out (Cannon *et al*. 2014), this probably does not occur (Korzeneiski & Rossiter, 2015). In the 64:128 s protocol, the contribution of cellular anaerobic glycolysis to ATP production became increasingly evident (Fig. 2) and pH_i_ fell during the exercise (unlike in the shorter intermittent protocols). This fall in pH_i_ during the work phase is consequent to a metabolic acidosis and associated lactate accumulation, and appeared to become more pronounced as the ∼30 min intermittent exercise progressed. Although this long duration intermittent protocol led to a more extreme cellular energetic strain, the intervening recovery bouts damped the magnitude of cellular energetic swings, thus prolonging exercise tolerance and increasing the volume of work accumulated (compared to continuous exercise at the same power output).

### Implications

Although the 64:128 s protocol was sustainable for the target duration and total accumulated work, the intramuscular and systemic metabolic responses suggest that participants were close to intolerance by the end of this protocol: peak ${\dot{V}}_{O_{2}}$ and PCr response were not different from continuous exercise (Fig. 3 and Table 2). This greatly contrasts the 16:32 and 32:64 s intermittent protocols, where systemic and intramuscular responses were of moderate and heavy intensity, respectively, and exercise could likely be sustained far beyond the ∼30 min protocol. This was despite accumulating the same amount of total work, in the same amount of time, in all three intermittent protocols. Thus, during shorter duration work:recovery bouts, the internal and external bioenergetic homeostasis was better maintained, and intensity reduced, during work‐ and duration‐matched exercise.

The dissociation between power output and bioenergetic function may have important implications for understanding the variability in the physiological adaptations to intermittent exercise, or for tailoring intermittent exercise training protocols to target specific physiological adaptations. Although intermittent exercise can be superior to traditional continuous moderate‐intensity exercise for increasing whole‐body V˙O2 max , muscle oxidative capacity, angiogenesis or stroke volume (Kemi *et al*. 2005; Helgerud *et al*. 2007; Wisløff *et al*. 2007; MacInnis *et al*. 2017), other studies find no difference between the training interventions (e.g. Gibala *et al*. 2006; Burgomaster *et al*. 2008; Bartlett *et al*. 2012; Ellingsen *et al*. 2017). In instances of no difference between training approaches, the specific power and intermittent duration of the protocols used may not optimize the intramuscular energetic response to promote remodelling (assuming intramuscular biogenic adaptations are a goal of the training). Our data emphasize that, for example, intermittent exercise at 60% of peak aerobic power with a 60:60 s work:recovery duration probably induces a greater intramuscular bioenergetics homeostatic challenge than a protocol using 110% of peak aerobic power and a 15:15 s work:recovery intermittent protocol (Gayda *et al*. 2012).

Given the protocol dependence of the dissociation between the external power and intramuscular metabolic strain, intermittent exercise allows a greater mechanical power to be achieved during training interventions than would otherwise be possible during continuous exercise. This dissociation also ameliorates the ventilatory demands and perceived exertion from the metabolic requirement of this mechanical power that would otherwise be associated with high‐intensity exercise. Given the mechanical load on the skeletal muscle is, in and of itself, an important signal for driving skeletal muscle adaptation in the absence of a metabolic challenge (Hellsten *et al*. 2008; Høier *et al*. 2010), our data have implications for the optimization of rehabilitation in clinical populations. For example, a high relative power with short work:recovery durations would provide a high mechanical strain without the associated metabolic response. This allows for a functional improvement by overcoming pathological pulmonary or cardiovascular system limitations that would normally limit the external power output achieved during training. Conversely, the relative importance of metabolic signalling (e.g. by AMPK) in driving beneficial muscular adaptations means that the stimulus during short work bouts may not be sufficient to optimize the training stimulus. Thus, our findings of dissociating muscle metabolic responses from mechanical power require further systematic investigation in relation to intermittent exercise training protocols.

### Conclusions

Performing dynamic knee‐extensor exercise at the same high‐intensity power intermittently reduces the O_2_ cost and the intramuscular metabolic strain of performing the same power during continuous exercise. Mean intramuscular ATP production rates are not different in intermittent and continuous exercise at the same power output. Despite this, pulmonary ${\dot{V}}_{O_{2}}$ increases less during short intermittent exercise (work:recovery 16:32 s) than during longer intermittent exercise (32:64 s or 64:128 s) and PCr contributes relatively more to ATP production during short *vs*. longer intermittent or continuous exercise. The latter suggests proportionally greater stored O_2_ usage during short work:recovery intermittent exercise than longer intermittent or continuous exercise. In addition, as the intermittent exercise work bout duration increases towards becoming continuous, relative ATP production relies increasingly upon anaerobic glycolysis and oxidative phosphorylation and less upon PCr breakdown. Our data are also consistent with ${\dot{V}}_{O_{2}}$ kinetics being an important determinant of exercise tolerance, through the rate of O_2_ deficit accumulation; even during intermittent exercise. The extent that we could dissociate power output and exercise intensity was greatest at the shortest work:recovery durations and was observable within the intramuscular bioenergetics.

## Additional information

### Competing interests

The authors declare that they have no competing interests.

### Author contributions

MD and CF conceived the study. All authors contributed to the design of the study. MD, GJK and CF collected the data. MD, GJK and CF analysed the data. All authors contributed to the interpretation of the data. MD and CF prepared the first draft of the manuscript. All authors critically reviewed and approved the final version of the manuscript submitted for publication, and agree to be accountable for all aspects of the work. All persons designated as authors qualify for authorship, and all those who qualify for authorship are listed.

### Funding

This research was supported by BBSRC UK (BB/I00162X/1 & BB/I001174/1) and a University of Leeds International Research Collaboration Award.
